# The ComP-ComA Quorum System Is Essential For “Trojan horse” Like Pathogenesis in *Bacillus nematocida*

**DOI:** 10.1371/journal.pone.0076920

**Published:** 2013-10-09

**Authors:** Xidan Deng, Yunxia Tian, Qiuhong Niu, Xiao’e Xu, Hui Shi, Hanbo Zhang, Lianming Liang, Keqin Zhang, Xiaowei Huang

**Affiliations:** 1 Laboratory for Conservation and Utilization of Bio-Resources and Key Laboratory for Microbial Resources of the Ministry of Education, Yunnan University, Kunming, Yunnan, PR China; 2 Tianjin Institute of Industrial Biotechnology, Chinese Academy of Sciences, Tianjin, PR China; 3 School of Life Science and Technology, Nanyang Normal University, Nanyang, Henan, PR China; Rockefeller University, United States of America

## Abstract

*Bacillus nematocida* B16 has been shown to use “Trojan horse” mechanism in pathogenesis that has characteristics of “social” behavior. The ComP-ComA system, a conserved quorum sensing system in the genus *Bacillus*, functions in many physiological processes including competence development, lipopeptide antibiotic surfactin production, degradative enzyme production and even some unknown functions. Here we investigated the requirement of ComP-ComA system in *B. nematocida* B16 for its pathogenicity against nematodes. The Δ*comP* mutant displayed deficiencies in attracting and killing nematodes, due to the absence of attractive signal molecules and the decreased expressions of virulence factors, respectively. Contrarily, a complemented *comP* mutant at least partially resumed its pathogenicity. Our data from transcriptional analysis further confirmed that this signaling system directly or indirectly regulated the expressions of two major virulence proteases in the infection of *B. nematocida* B16. Bioinformatics analyses from comparative genomics also suggested that the potential target genes of transcription factor ComA were involved in the processes such as the synthesis of attractants, production of extracellular degradative enzymes and sortase, secondary metabolites biosynthesis, regulation of transcription factors, mobility, as well as transporters, most of which were different from a saprophytic relative *B. subtilis* 168. Therefore, our investigation firstly revealed that the participation and necessity of ComP-ComA signaling system in bacterial pathogenesis.

## Introduction

*Bacillus nematocida* strain B16 has been isolated from a soil sample in Yunnan province in China, and is pathogenic to free-living nematode *Panagrellus redivius* as well as the plant parasite nematode *Bursaphelenchus xylophilus* [1]. It has also been shown to lure nematodes to their death by a “Trojan horse” mechanism. The bacterium *B. nematocida* produces potent volatile organic compounds (VOCs) that are much more attractive to worms than those from ordinary dietary bacteria, and successfully traps its hosts. Then the pathogenic factors, mainly including an extracellular alkaline serine protease Bace16 and a neutral protease Bae16, are responsible for death of nematodes [2]. In this type of multistep infection process that has characteristics of “social” behavior, a precise “combat command system” should be required to modulate these processes in infection, but remains unknown.

In bacteria, quorum sensing (QS) enables an individual bacterial cell to sense other bacterial cells, and in response, synchronously switches specific sets of genes. It contributes to the ability of bacterial population to instigate a collective behavioral change to environmental challenges. QS system typically involves the activation of a sensor or response regulator by small signal molecules. The signal molecules are synthesized by the specific genes, and then after modification they diffuse freely across the cell membranes or are actively transported out of the cell. Once the concentration of secreted signal molecules has reached a threshold level, they are detected by cognate sensor proteins that either transduce the signal to downstream transcriptional regulators, or themselves function as transcriptional regulators, to mediate changes in gene expression [3]. Till now, at least three QS signaling systems have been identified based on the different types of signal molecules, including N-acylhomoserine lactone (AHL) in Gram-negative signaling systems [4], autoinducing peptides (AIPs) in Gram-positive signaling systems [5], and AI-2-type interspecies signaling systems [6]. Among them, the QS systems that depend on the signal molecules of oligopeptides to trigger two component phosphorelay is only used by Gram-positive bacteria, and requires further elucidated [7]. ComP-ComA is one of this type QS that has been well investigated in the model species *B. subtilis*168. In this signaling system, the kinase ComP has been suggested as the only receptor for the signaling molecule ComX, and that ComA is the only transcription factor activated directly by ComP in *B. subtilis*168 [8]. So, when bacterial cells reach a high cell density, the ComX peptide signaling leads to ComA-regulated processes such as competence development [9], lipopeptide antibiotic surfactin production [10,11], degradative enzyme production [12], as well as other unknown functions [13].

Here, in our investigation whether this QS controls the pathogenic process in *B. nematocida* B16, our experimental evidences suggest the involvement of ComP-ComA in synthesis of the attractive signals and production of the two virulence proteases. Additionally, bioinformatics analyses demonstrate that the candidate target genes regulated by ComP-ComA in *B. nematocida* B16 were quite different from those predicted in a saprophytic bacterium *B. subtilis* 168, one of the closest relatives of *B. nematocida* in the *Bacillus* genus but with almost no nematocidal activity (Figure S1 in File S1). Those potential target genes in *B. nematocida* B16 included a variety of pathogenic genes. Thus, our investigation is the first report about the roles of signaling system ComP-ComA in the infection of bacterial pathogens.

## Material and Methods

### 1: Ethics Statement

No specific permits were required for the described field studies. No specific permissions were required for these locations/activities. The location is not privately-owned or protected in any way.

### 2: Bacterial strains, plasmids, growth conditions

The strains and plasmids used in this study are listed in Table S3 in File S1. The primers used in this study are listed in Table S4 in File S1. Luria-Bertani (LB) medium was commonly used for the cultivation of bacteria except when specially mentioned. *Escherichia coli* DH5α was used as the host strain for the construction and maintenance of plasmids. Antibiotics were used at the following concentrations: 5mg/ml chloramphenicol, 0.5mg/ml erythromycin and 10mg/ml kanamycin, respectively.

### 3: Genetic manipulation

The integration vector for Gram-positive bacteria *p*CP115, obtained from the Bacillus Genetic Stock Center (BGSC), was used to construct Δ*comP* mutant in *B. nematocida* B16 through homologous recombination. The primers, *comP*1 and *comP*2 with the restrictive endonucleases of *Eco*RI and *Pst*I, were used for amplifying the partial *comP* encoding gene and produced a 210 bp fragment. The fragment was then inserted into the integration vector *p*CP115, and then the recombinant plasmid *p*CP115Δ*comP* was selected and amplified in *E. coli* strain DH5α. The competent cells of *B. nematocida* were prepared and transformed with *p*CP115Δ*comP* according to the protocols supplied by BGSC. A clone carrying a single-crossover mutation of *comP* was obtained by selection on LB agar medium containing 5µg/ml chloramphenicol. PCR analysis with primers *comP*3 and *comP*4 further confirmed the knockout mutant, in which the two primers were designed based on the chromosomal DNA sequence and the downstream sequence of the integration vector *p*CP115, respectively. Thus the corresponding DNA fragment amplified by PCR would be obtained only if *p*CP115Δ*comP* had inserted into the target locus of the chromosome.

Full length gene of *comP* was amplified via PCR, and digested with HindIII and SphI at primer- incorporated restriction sites, and inserted into a HindIII/SphI -digested pDG148 vector to obtain the plasmid for a complemented *comP* mutant.

### 4: Nematocidal activity assays

Pieces of autoclaved cellophane paper were used to cover the agar plates (2%) containing a low-nutrient mineral salt medium, and then bacteria were inoculated onto the cellophane paper and incubated at 37°C for 24 hrs. The tested nematodes were placed in the middle of the plate. The numbers of live nematodes were counted right after inoculation and every 12 hrs afterwards. Negative controls included the non-pathogenic bacteria *E. coli* strain OP50 as well as no bacteria under the same conditions.

### 5: Protease assays

The bacterial strains were grown in LB medium at 37°C, and then the supernatants were collected at different times. The protease activity was assayed by a modified caseinolytic method. One unit (U) of protease activity was defined as the amount of enzyme needed to hydrolyze the substrate and produce 1 mg tyrosine under the assay conditions.

### 6: RT-qPCR analysis

The total RNA was isolated using RNA extracting kit (Tiangen, China) following the treatment of DNaseI to avoid DNA contaminant. RNAclean Kit (BioTeck, China) was then employed to further purify the total RNA. RNA concentration was determined by measuring absorbance at 260 nm using a UV spectrophotometer. After random-primed cDNAs were generated, qPCR analysis was performed with SYBR Green JumpStart Taq Ready Mix for qPCR kit (Sigma-Aldrich Co) following manufacturer’s instructions. The partial sequence of 16s rRNA amplified by primers O1 and O2 was used for an internal control; E1 and E2 were primers for *nprE*; J1 and J2 were primers for *aprE*. PCR amplification followed by 40 cycles of 94°C for 30 s, 60°C for 31 s, 72°C for 40 s on ABI PRISM 7000 Real-Time PCR.

### 7: Predicting the binding sites of ComA in promoters of *B*. *Nematocida* B16

Genome sequences of *B. subtilis* (NC_000964) and *B. nematocida* with the annotation information were obtained from NCBI. Orthologous between *B. subtilis* and *B. nematocida* were obtained by using orthomcl [14]. Operons in the two genomes were predicted by DOOR: Database of prokaryotic operons [15].

Before predicting the potential binding sites of transcription factor ComA, we excluded genes with upstream intergenic regions of less than 30 bp to reduce potential noise though a few binding sites for transcription factors had been described to exist in coding regions. Next, the intergenic regions of each gene were uploaded to DBTBS (Database of *B. subtilis* Transcription factors) (http://dbtbs.hgc.jp/) [16] to find ComA binding sites by a Weight Matrix search (1% threshold). If the first gene in an operon contains an upstream ComA binding motif, we consider all genes in this operon as regulated by this transcription factor.

### 8: SPME-GC/MS analysis

A headspace solid-phase microextraction (SPME) method in combination with GC/MS was employed for quantification of the major volatile organic compounds (VOCs) of the samples. The tested strains, including *B. nematocida* B16 and Δ*comP* mutant, were cultivated at 37°C over 24 hrs. 5ml samples (the bacterial cultures) were placed in 15ml vials with a magnetic stirrer and extracted with the extraction head (CAD-PDMS 75µm, Supelco). The fiber was exposed to the headspace above the sample for 65°C for 60 min followed by inserting into the GC injector of Clarus 500 GC/MS System (PE Co, Waltham, Massachusetts, USA) and desorbing at 250°C for 2 min. DM-5 column (DM-5 30m x 0.32mm x 1µm) (Chrompack, Mid-delburg, Netherlands) was used in our GC/MS analysis. The initial oven temperature was 50°C, held for 2 min, ramped 4 °C/min to 180°C and then 6 °C/min to 280°C, and held for 10 min. The volatile components were detected by mass spectrometry with electron impact ionization at 70 electron volts, with a continuous scan from mass to charge ratio (m/z) 35 to 550. Compounds were identified by matching the mass spectra with standards in the NBS 2005 library and Nist 2005 library. The blank medium was used as negative control.

### 9: β-galactosidase assay

Plasmid pIS284 (a gift from Dr Mitsuo Ogura, Japan), containing the lacZ gene was used for constructing reporter vectors for transformation. To construct pA1, pA2, pA3, pA4, pA5, pN1, pN2, and pN3, the extracted genomic DNA from *B. nematocida* B16 was used for PCR amplification with the pairs of primers A1 and ARV, A2 and ARV, A3 and ARV, A4 and ARV, A5and ARV, N1 and NRV, N2 and NRV, N3 and NRV, respectively (Table S4 in File S1). The DNA fragments were then digested with *Hind*III and *Eco*RI, and inserted into the similarly digested pIS284 plasmid. Finally, plasmids pA1, pA2, pA3, pA4, pA5, pN1, pN2, and pN3 were transformed into *B. nematocida* B16, resulting in the strains A1, A2, A3, A4, A5, N1, N2, and N3, respectively (Table S3 in File S1).

Specific β-galactosidase activities were determined from growing liquid cultures in LB medium at 12h, 24h, and 36h. β-galactosidase was assayed using ο-nitrophenyl-β-D-galactopyranoside as the substrate and is reported in Miller units.

### 10: Electrophoretic mobility shift assay (EMSA)

A 26bp DNA fragment harboring the *bae16* promoter region between -185 to -160 was labeled by biotin as a probe. The EMSA was performed using a Light Shift Chemiluminescent EMSA Kit (Pierce, Rockford, IL, USA) according to the manufacturer’s instructions. The DNA binding reaction was carried out by the addition of 30ng purified ComA protein that had been heterologously expressed in *E. coli* BL21 using the plasmid *p*ET30. After incubated at 30°C for 30 min, the samples were then loaded on a pre-run 6% (w/v) non-denaturing polyacrylamide gel. The gel was run at 100 V in 0.5×TBE buffer for 2h at 4°C. For the competition assay, a 50- and 100-fold excess of unlabeled probe was added to the binding reaction mixtures.

## Result

### 1. The involvement of ComP-ComA in the synthesis of attractants

**Figure 1:**
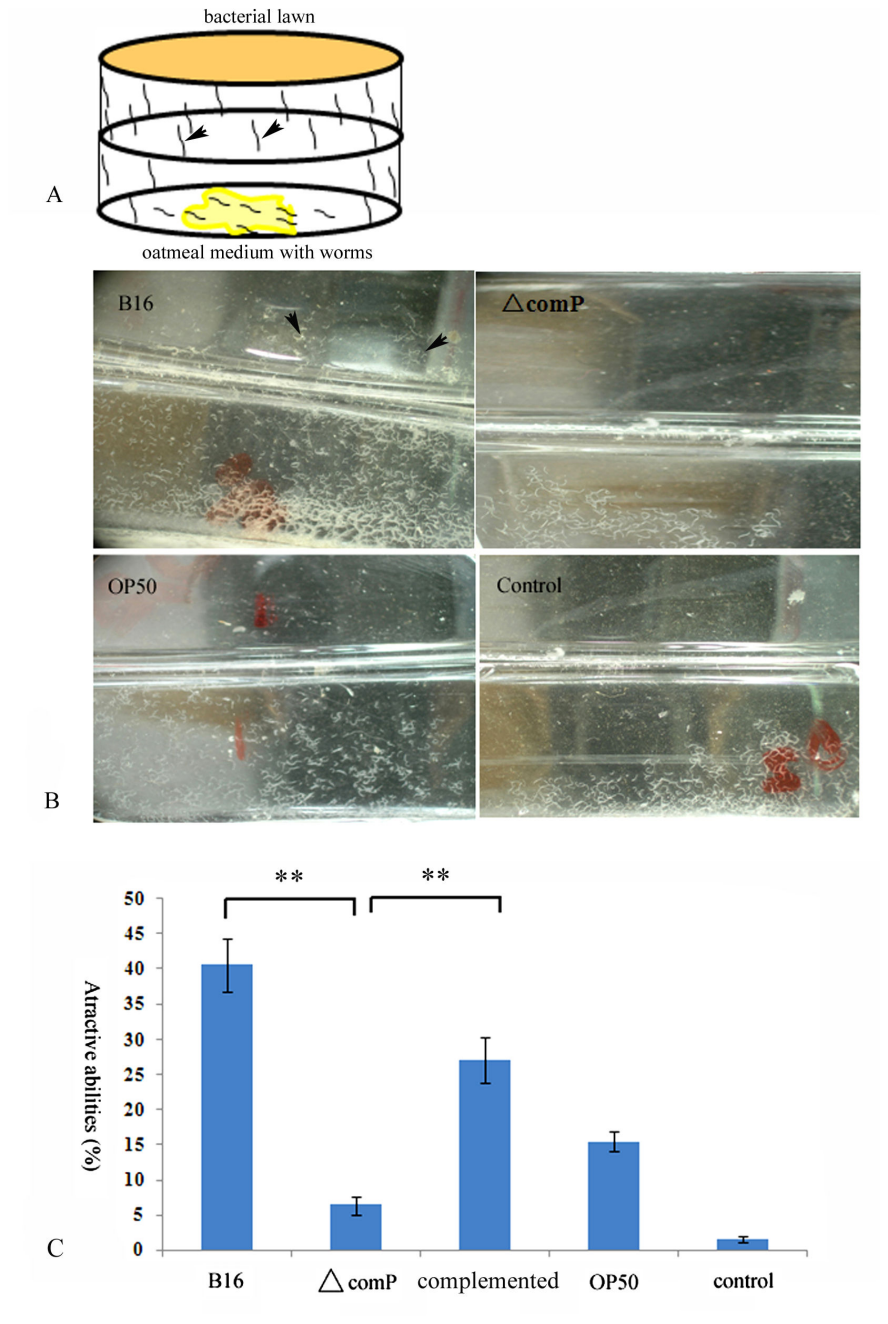
Comparison of the attractive abilities for nematodes in different strains of *B. nematocida*. (A) Schematic diagram of inverted Petri dish attractant experiment. In this method, the movement of nematodes on the plates was assessed by counting the number of nematodes on the upper lid using a stereomicroscope. (B) Assays to the attractive abilities in the wide type strain, Δ*comP*, the complemented *comP* mutant, *E. coli* OP 50 and blank medium with the method of inverted Petri dish. Arrowheads indicate the appearance of the nematodes that climbed towards the upper dish. ** represented significant difference by t-test (*P* < 0.01). (C) Graphical representation of the percentage of nematodes that climbed towards the upper dish within 12 hrs.

To investigate the involvement of ComP-ComA in regulating the synthesis of the VOCs that had been suggested the prerequisites for successful infection against nematodes [2], the mutants of genes *comP* and c*omA* were constructed to testify their effects on nematitoxic activities respectively. However, the mutant of Δ*comA* was not obtained like Δ*comP* strain though the same methods were used, might due to its lethality. Then, the attractive capabilities for nematodes were compared between Δ*comP* mutant and the wild strain by using the method of inverted Petri dish. Our data demonstrated that the disruption of gene *comP* made the bacterium lost most of its attractive capabilities for nematodes, which was similar to the result from the negative control of blank medium (Figure 1A). Quantity analysis within 12 hrs revealed that Δ*comP* strain lured only 6.45±1.30% *C. elegans* migrating upwards and reaching the upper Petri dishes that contained the tested bacterial lawn, with 40.61±3.74% worm migrated towards the wild type strain, 15.47±1.38% towards *E. coli* OP 50 (one kind of food for nematodes), and 1.58±0.42% in blank medium (Figure 1B). When complemented the expression of *comP* in Δ*comP*, the phenotype was resumed with 27.16±3.21% worm migrating towards the upper lid (Figure 1B).

**Figure 2:**
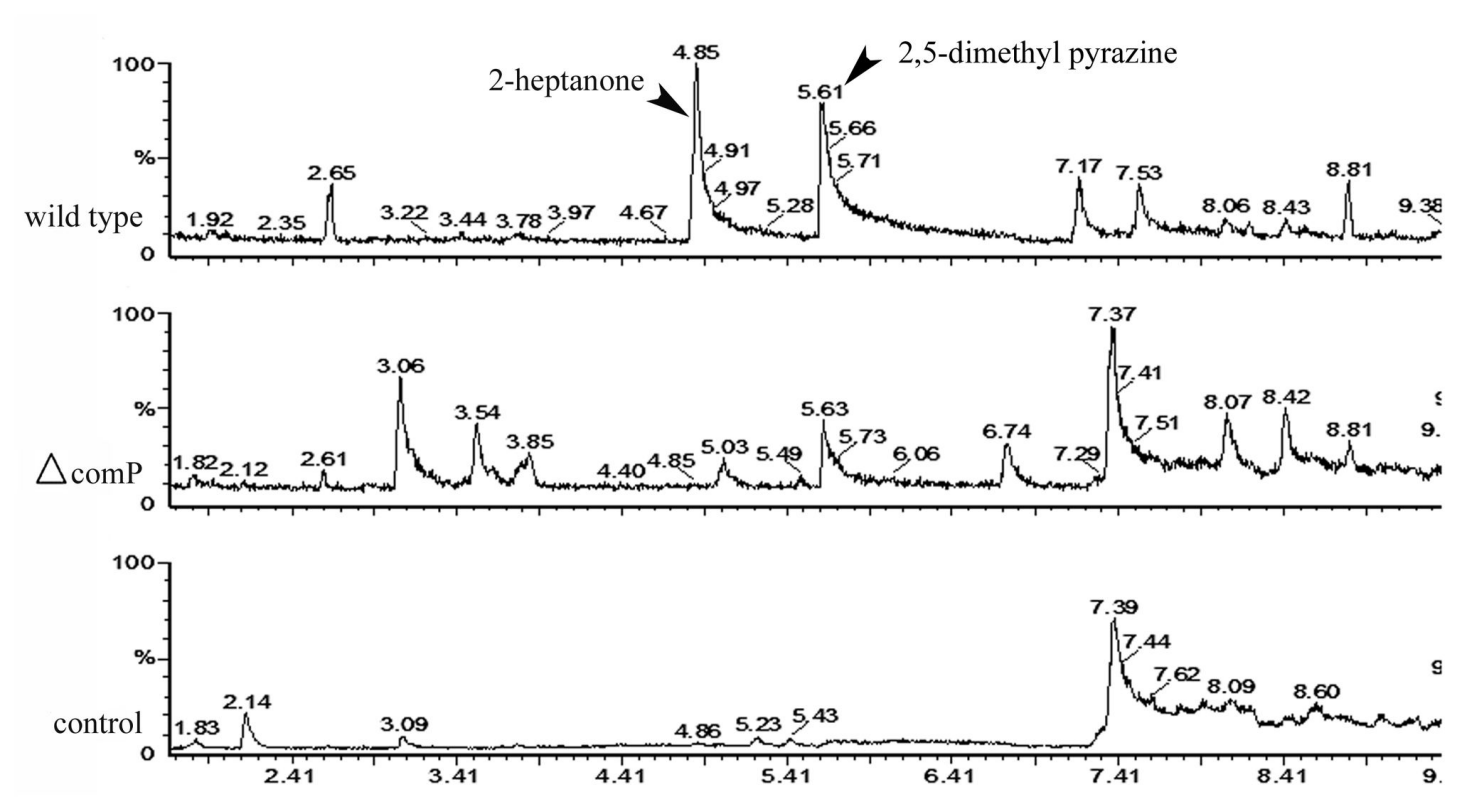
The changes of VOCs in Δ*comP* mutant were determined with SPME/GC-MS. Arrowhead represented the attractive signal molecules differently between the wide type strain and Δ*comP* mutant.

The changes in the production of attractive VOCs in Δ*comP* mutant were further detected. Compared to the wild type strain and the negative control of blank medium, VOCs in Δ*comP* mutant strain changed in both categories and quantities. Our results obviously showed disappearance of 2-heptanone (retention time 4.85 min) and decrease of 2, 5-dimethyl pyrazine (retention time 5.61 min), and either of the two molecules had been validated to be attractants for *C. elegans* at low concentrations [2,17]. We also observed appearance of several new peaks, such as furfural (retention time 3.06), butanoic acid 2-methy ethyl ester (retention time 3.54), 2-furanmethanol (retention time 3.85), and an uncertain compound (retention time 6.74), which were possiblely derived from the disordered biosynthetic pathway for VOCs (Figure 2).

### 2: The expressions of virulence proteases are regulated by ComP-ComA

**Figure 3:**
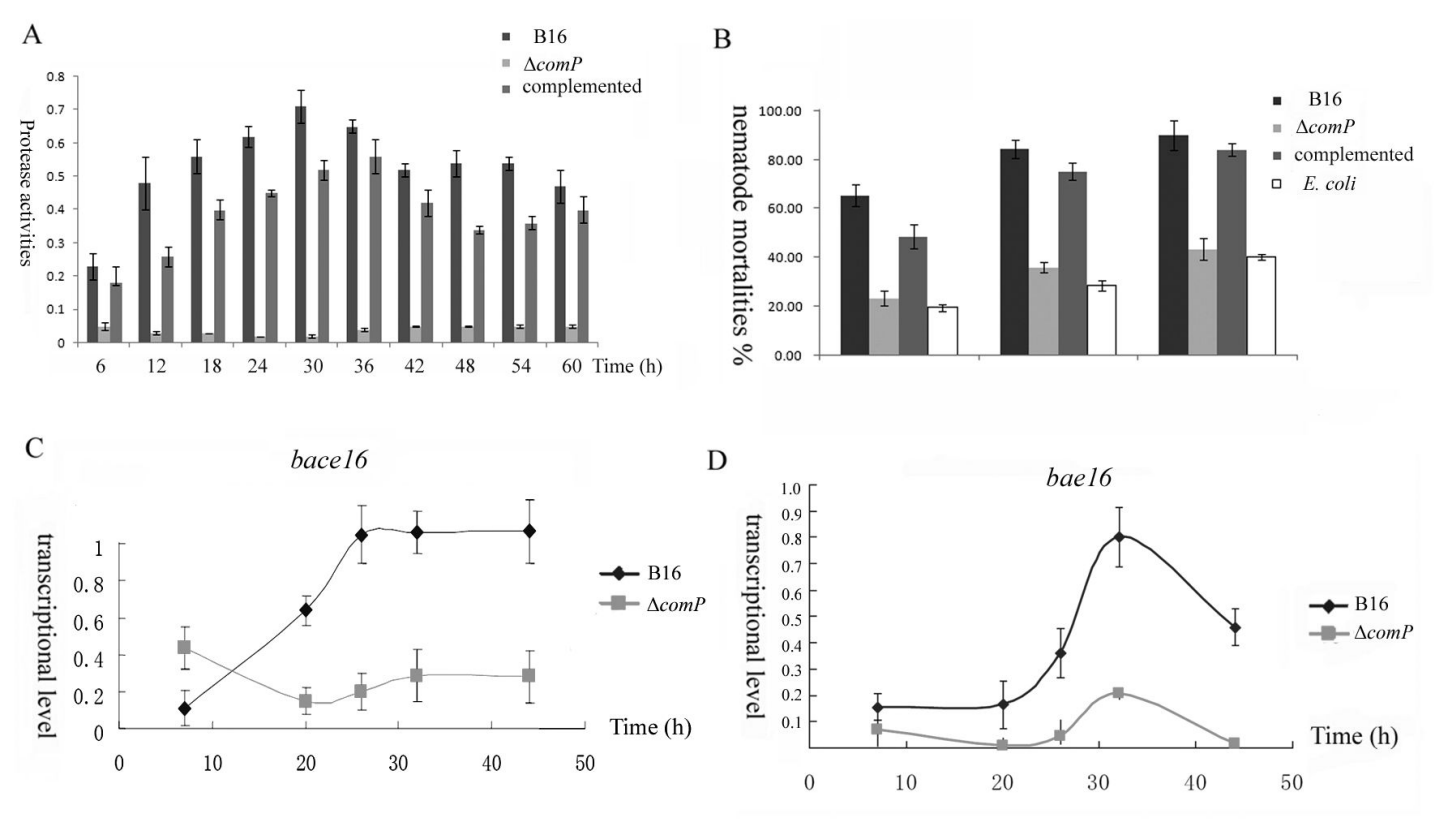
ComA-ComP regulated the expressions of virulence proteases *bace16* and *bae16*. (A) The protease activities were assayed using folin method in wide type strain, Δ*comP*, and the complemented *comP* mutant. (B) The mortalities of nematodes were determined in wide type strain, Δ*comP*, the complemented *comP* mutant, and the negative control *E. coli*. (C) and (D) Relatively transcriptional levels of the two virulence proteases *bace16* and *bae16* were detected by qRT-PCR, which had been normalized by the internal control of l 6s rRNA gene.

In an attempt to examine whether ComP-ComA could also impact nematotoxic activities, we detected the changes of protein hydrolysis and nematode mortalities in Δ*comP* as well as the complemented *comP* mutant. It was shown that the strain of Δ*comP* had lost the majority of its protease activities (Figure 3A) and remained less than half of nematocidal activities (Figure 3B) within our tested time points, suggesting the positive relationship between QS system and the expressions of virulence genes in the wild type strain. Similarly, either protease activity or nematocidal activity obviously restored in the complemented *comP* mutant (Figure 3A and B). With qRT-PCR, we directly detected the transcriptional levels of two genes *bace16* and *bae16* that had been described as the key virulence factors in our previous study [2]. Our experimental data demonstrated that, in wild type strain, the expression of two proteases was associated with cell concentrations and was enhanced during cell exponential growth. However, once the gene *comP* was knocked-out, their expressions decreased obviously and less than 30% proteases production was retained compared to the parental strain (Figure 3C and Figure 3D), suggesting that the ComP-ComA system positively controlled the expressions of virulence genes *bace16* and *bae16*.

### 3: Transcriptional analysis of two virulence proteases Bace16 and Bae16

**Figure 4:**
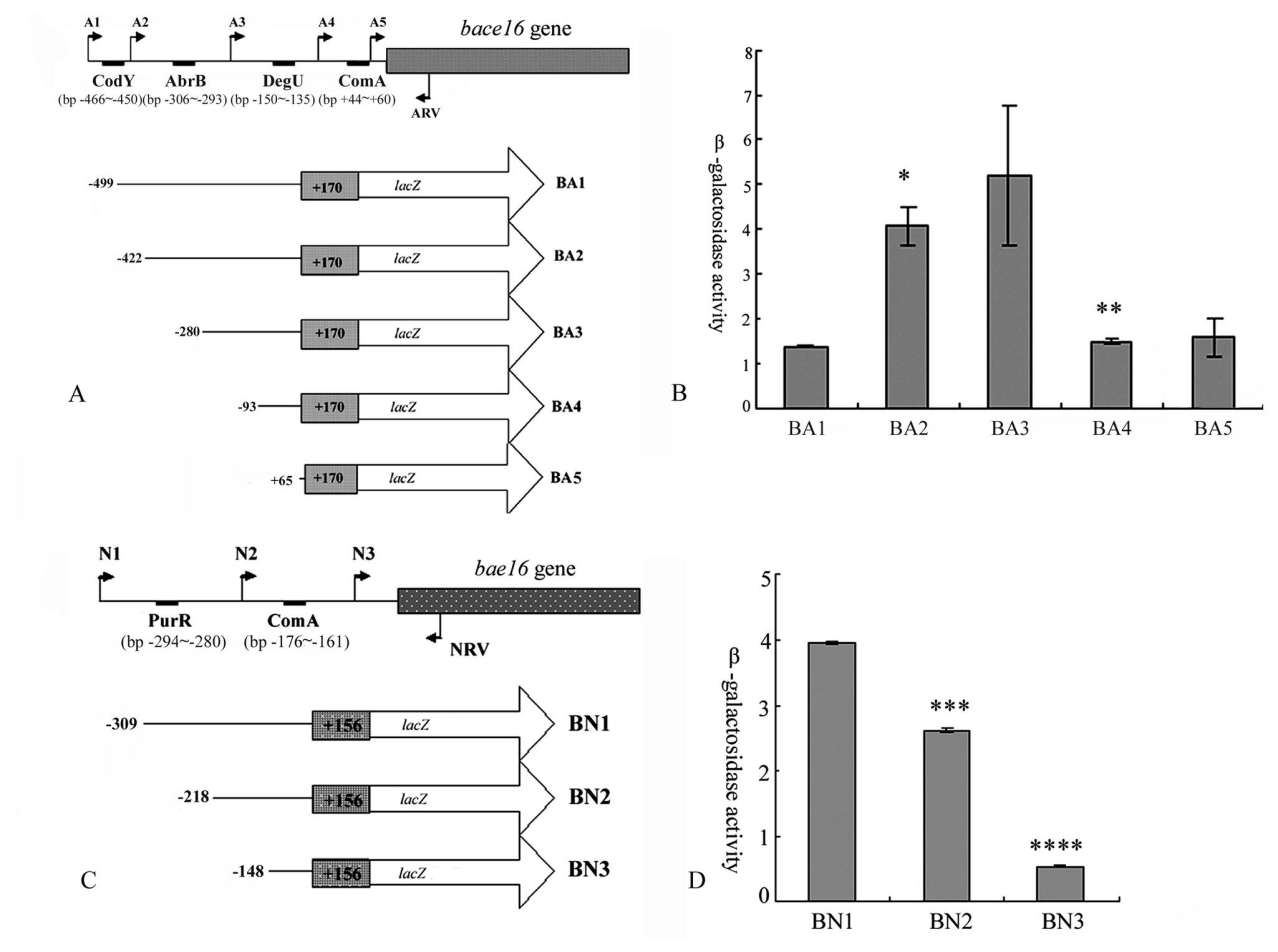
Mapping of the *bace16* and *bae16* promoter regions by 5’ deletions. (A) Schematic representation of the different *bace16::lacZ* fusions used in this study. The filled arrows indicate the primers used for generating the various reporter fusions (A1 to ARV), wheras the 5’ and 3’ end termini of the *bace16::lacZ* fusions are denoted with their nucleotide position relative to the initiator codon. The black oblongs indicate the binding motifs in the promoter of *bace16*, including CodY, AbrB, DegU and ComA. The derivative *B. nematocida* B16 carry the respective fusions are shown the below. (B) Expression of the series of bace16::lacZ fusions respectively. Cells were grown in LB medium at 37°C, and β-galactosidase activities were determined at 36h. (C) Schematic representation of the different *bae16::lacZ* fusions used in this study, which is similar to the *bace16::lacZ* fusions. (D) Assays to β-galactosidase activities in the series of bace16::lacZ fusions at 36h. * represented *P* < 0.05 by t-test; ** represented *P* < 0.01 by t-test.

Since the genes in synthetic pathway of VOCs have not been identified in the *Bacillus* genus, the transcriptional analysis within the promoter regions were performed in two virulence proteases. Furthermore, the cis-activating elements of *bace16* and *bae16* in *B. nematocida* B16 were predicted by DBTBS because *B. subtilis* is one of the closest relatives. With this method, the potential binding motifs of CodY, AbrB, DegU and ComA were found within the promoter region of *bace16* (*P* value<0.01%) (Figure 4A), with the binding motifs of PurR and ComA within the promoter region of *bae16* (*P* value<0.01%) (Figure 4C). Then, we determined the amino-acid sequence similarities of these trans-activating factors between the two species with CodY 99.23%, AbrB 97.87%, DegU 99.56%, PurR 94.01%, ComA 85.98%, along with predictions of three-dimensional protein structures based on homologous modeling (Figure S2 in File S1) confirmed validities of the above results.

In order to experimentally monitor the transcriptional regulations of the two virulence proteases, a series of reporter fusions containing the truncated promoter regions to *lacZ* were successfully constructed. Specifically, in the analysis of the promoter regions of genes *bace16* and *bae16*, five and three nested fragments with a common downstream end and variable upstream ends were fused to a promoter-less *lacZ* gene in pIS284 respectively (Figure 4A, 4C). After β-galactosidase activity in each strain was measured, it was demonstrated that strain BA2 had higher β-galactosidase activity than BA1 that contained an additional transcriptional motif of CodY (*p<0.01), but strain BA4 that missed the cis-activating element of DegU represented lower β-galactosidaseactivity than BA3 (**p<0.01) (Figure 4B). In the analysis to β-galactosidase activity within *bae16* promoter region, strain BN1 had higher activity than BN2 (***p<0.01), and strain BN2 had higher activity than BN3 (****p<0.01) (Figure 4D). The data meant that cis-activating element of CodY and DegU participated in the transcription of *bace16*; while PurR and ComA regulated the transcription of *bae16*.

### 4: Transcription factor ComA directly binds to the promoter of virulence protease Bae16

**Figure 5:**
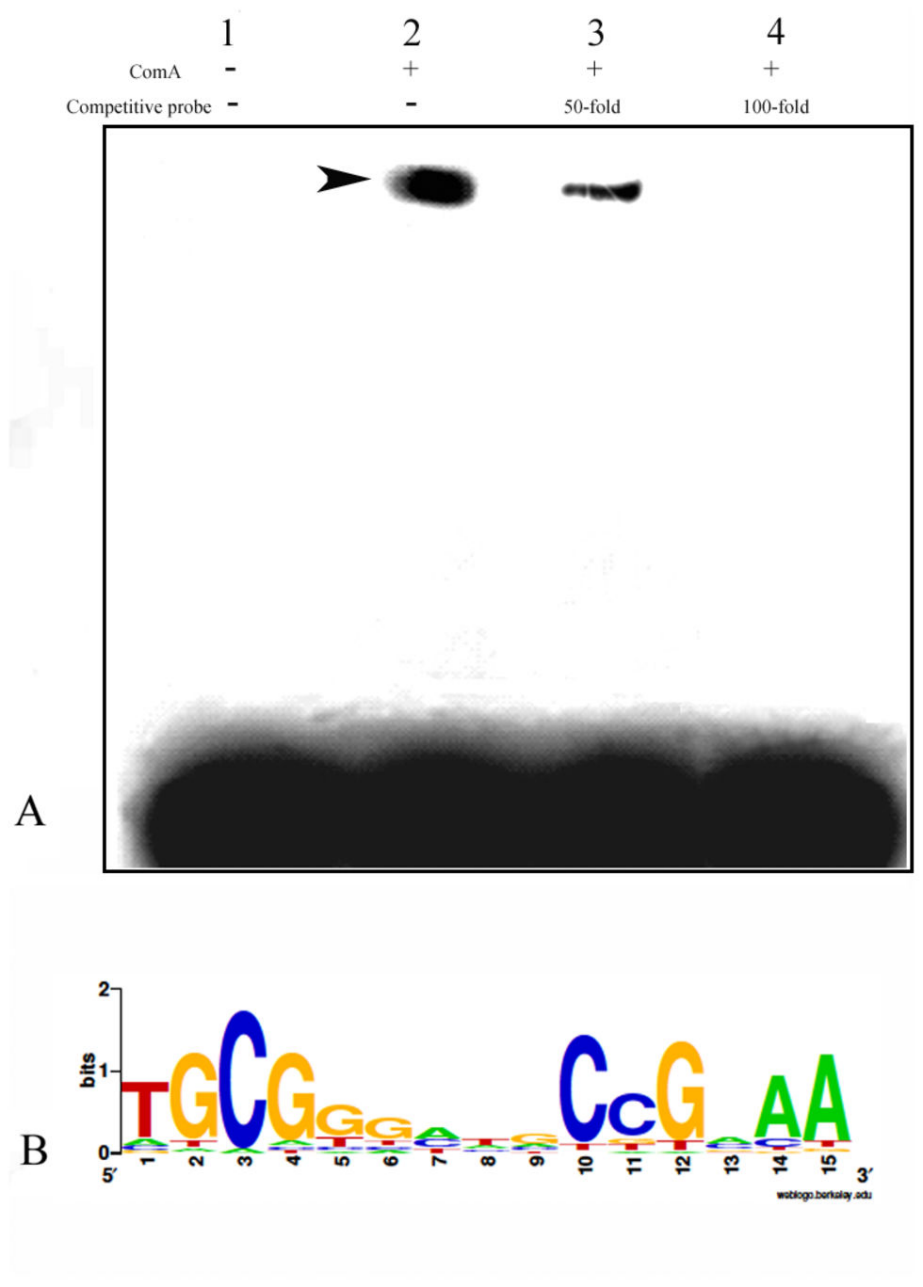
EMSA to the labeled DNA probe within *bae16* promoter. (A) The result from EMSA suggested interaction between the transcriptional factor ComA and *bae16* promoter. The arrow indicates the band of interest. (B) The predicted binding motifs of ComA in *B. nematocida* B16.

The experimental data above all illustrated that trans-activating factor ComA directly regulated the transcription of gene *bae16*. Next, we further determined whether the transcription factor ComA of *B. nematocida* B16 indeed interacted with the potential binding motif with the method of EMSA. After heterologously expressed the protein ComA of *B. nematocida* B16 and purified our target protein (Figure S3 in File S1), EMSA was performed using the labeled DNA probe harboring the predicted binding motif of ComA. It was demonstrated that our probe could bind to ComA. But when adding into 50- and 100-fold excess of competitive cold probes, the retarded band bleached and even completely disappeared (Figure 5A), which suggested that the transcription factor ComA interacts with the potential binding motif within the *bae16* promoter.

### 5: Characterization of potential target genes regulated by ComA within genome

**Figure 6:**
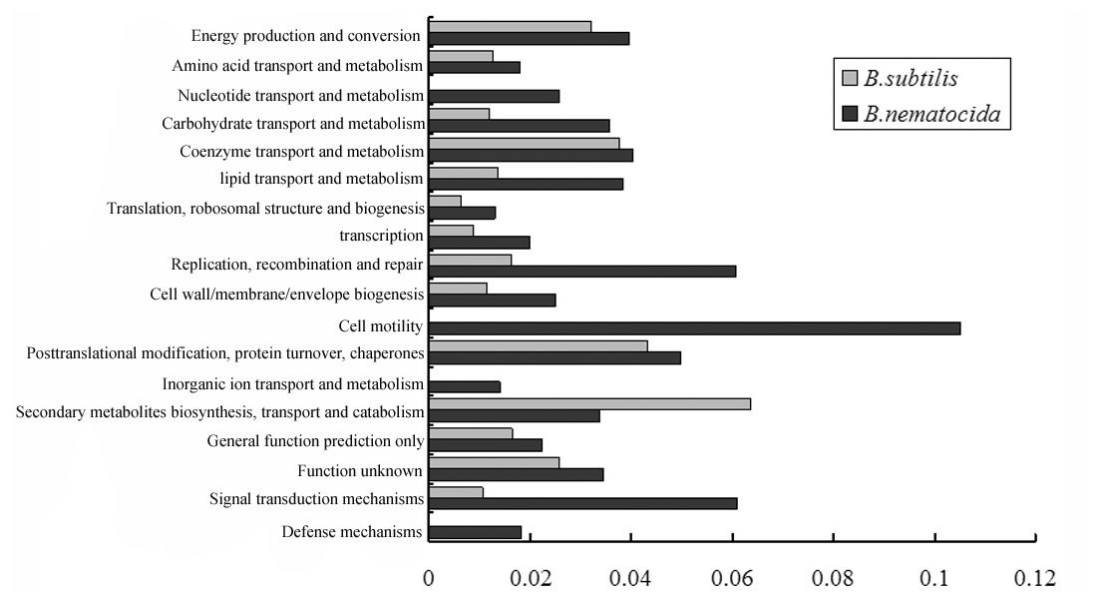
Comparative display of the COG occurrences of ComA regulated genes in *B. nematocida* B16 over those in *B. subtilis* 168. The Y-coordinate represents the percentage of the gene occurrences in given COGs, using genomic COG as background.

To further understand the relationship between ComP-ComA regulation network and virulence in this bacterium, we predicted the potential target genes that had at least one upstream ComA binding motif throughout the *B. nematocida* B16 genome, and analyzed the evolutionary trajectory of ComA-binding elements in its target genes. Our result suggested that altogether 126 genes within 72 operons contained the upstream ComA binding sites in *B. nematocida* B16 (Table S1 in File S1). Their functions included transcription factors, transporters, hydrolyses, oxidoreductases, cell motility, cell wall/membrane/envelope biogenesis, secondary metabolites biosynthesis and so on. Comparing the target genes with those in *B. subitilis* 186, one of the nearest neighbors to our tested strain but with little nematocial activity, it was shown that except for several conserved genes, such as degQ, srfAA, phrA, rapA, rapC, ywqG, more than 80% of target genes in the two species had no overlap, suggesting great divergence had happened in the QS system between the two organisms (Table S2 in File S1). COG category also illustrated several interesting differences between those target genes in the two species, especially signal transduction mechanisms, replication, recombination and repair, carbohydrate transport and metabolism, lipid transport and metabolism, transcription, cell wall/membrane/envelope biogenesis, translation, ribosomal structure and biogenesis with their gene number ratios more than 2 fold in *B. nematocida*. Additionally, ComA in *B. nematocida* regulated the genes involved in defense mechanisms, cell motility, inorganic ion transport and metabolism, nucleotide transport and metabolism uniquely (Figure 6). Based on the differential genes regulated by ComP/ComA signaling system, we further analyzed their association with pathogenicity in bacterium *B. nematocida* B16.

#### Synthesis of attractants to nematodes

Since most of the confirmed VOCs belong to aromatic compounds and ketones based on our previous report [2], we here analyzed the related KEGG pathways involved in attractants synthesis and compared the pathways potentially impacted by ComA. The synthesis of these attractants contained nine kinds of pathway, including toluene degradation, aminobenzoate degradation, ethylbenzene degradation, tryptophan metabolism, phenylalanine, tyrosine and tryptophan biosynthesis, benzoxazinoid biosynthesis, propanoate metabolism, synthesis and degradation of ketone bodies. Meantime, all of the genes targeted by ComA in *B. nematocida* comprised 34 pathways and four of them overlapped with attractants synthesis pathways, which are also supported by the data from production of VOCs in Δ*comP* mutant.

#### Sortase

Sortase is defined as a group of prokaryotic enzymes that catalyze the assembly of pilins into pili, or the anchoring of pili to the cell wall [18]. They act as both proteases and transpeptidases [19]. The pili endproducts often make the bacteria more virulent due to their increased adherence to host cells [20] or biofilm formation [21]. We found that the gene *YwpE*, a sortase, was controlled by ComA in *B. nematocida*.

#### Extracellular degradative enzymes

In many organisms, the production of extracellular degradative enzymes is controlled by the QS system [22]. This probably reflects the need to accumulate a sufficient extracellular concentration of the enzymes to degrade enough nutrients or to prepare for infection. Although microarray data showed that the ComP-ComA system in *B. subtilis* regulated some extracellular enzymes such as extracellular lipase *lip*, pectate lyase *pel* [11], both *bae16* and *bace16*, two of the most important virulence factors in *B. nematocida*, do not contain upstream ComA binding sites in *B. subtilis*. But our experimental data above revealed the involvement of ComA in regulating both the neutral metalloprotease *bae16* and the serine protease *bace16*. In addition, two metal-dependent proteases *gcp* and *ydiC*, and another peptidoglycan hydrolase with signal peptide *yqiI*, contained putative binding motifs of ComA in *B. nematocida*.

#### Drug/antibiotic transporters and amino acid, polysaccharide transporters

Antibiotic efflux pumps in prokaryotic cells can protect cells from exogenous, diffusible molecules by both extruding poorly diffusible or toxic endogenous molecules and targeting modification of antibiotic-inactivating enzymes [23]. It has been revealed that mutants of these effluxes have significantly reduced invasiveness [24]. Additionally, the positive relationship between antibiotic effluxes and QS system has also been suggested. For example, the auto-inducer 3-oxo-C12-homoserine lactone, which plays a key role in *Pseudomonas aeruginosa* pathogenesis, is regulated by the MexAB–OprM efflux system [25,26]. Three drug/antibiotic transporter genes *yojI*, *blt*/*norA*, and *ykfF* were predicted to be regulated by ComA uniquely in *B. nematocida* B16.

Amino acid transporters are necessary to pathogenic life since bacteria uptakes proteins from hosts. Pathogenic *Bacilli* like *B. thurengensis* or *B. cereus* contain more abundant amino acid utilization genes and amino acid transporters than saprophytic *B. subtilis* [27]. The predicted target genes directly regulated by ComA in *B. nematocida* included two amino acid transporters *ydaO* and BN_3613, as well as a polysaccharide transporter BN_0223.

#### Secondary metabolites biosynthesis

Three secondary metabolite biosynthesis genes were regulated by ComA directly in *B. nematocida*, including *srfAA*, *yrhH*, and *yuxO*. The product of srfAA operon is needed for the production of lipopeptide antibiotic surfactin that displays virulence to other bacterial species [28]. Another uncharacterized gene *yuxO*, a candidate gene involved in catabolism of aromatic compounds, might be associated with the synthesis of attractants in *B. nematocida*.

#### Transcription factors

ComA in *B. nematocida* also targeted six transcription factors or systems, including ComA system itself, degQ/degU system, citT/citS system, *yojH*, *ydhC*, *ywhA* and *yybT*. By interacting with other transcription factors, the ComP-ComA system could enhance its ability to regulate the infection process. For example, ComA in *B. amyloliquefacien* has been reported to regulate the expression of DegQ-DegU system, and therefore influenced the production of the virulence proteases as well as secondary metabolites such as surfactin, fengycin, and bacillomycin D (Figure S3 in File S1) [29,30].

#### Mobility

MotA probably functions as a transmembrane proton channel and as part of the flagellar motor [31]. The investigation in the pathogenesis of *Dickeya dadantii* suggested that mutants of *motA* had the most significant decrease in the swimming ability and virulence to certain hosts [32]. Therefore, the relationship between ComP-ComA system and motility in *B. nematocida* seems not an incidental event-

## Discussion

QS system in prokaryotic bacteria is a specific transcriptional system to coordinate gene expression on a population-dependent manner. It functions in processes such as virulence, sporulation, genetic transfer, and production of nisin or other secondary metabolites. In pathogenesis of bacterial pathogens against hosts, agr (accessory gene regulator) is central to virulence gene regulation in *Staphylococcus aureus*. It has been well described that intracellular survival of pathogen in epithelial and endothelial cells, biofilm development, and three major exotoxin classes (a-toxin, PVL and the PSMs) are all regulated by this QS system [33-35]. Meantime, *fsr* quorum-sensing system in *Enterococcus faecalis* that is highly homologous to *agr* in *S. aureus* positively activates the expression of two extracellular virulence-related proteins or some other pathogenic factors. Thus, the *fsr* mutants have shown the attenuated abilities to kill its different hosts including nematodes, mice, and rabbits [36,37]. The transcription factor PlcR in QS system of PlcR-PapR, regulates the extracellular virulence factors including phosphatidylinositol-specific phospholipase C, phosphatidylcholine-preferring phospholipase C, and haemolytic or non-haemolytic enterotoxin in *B. cereus* group [38,39]. Collectively, QS signaling systems control bacterial pathogenesis in bacterial species.

In our nematotoxic strain of *B. nematocida* B16, we also scanned the homologs of the QS genes that had been suggested in bacterial pathogenesis such as *agr*, *fsr* and *plcR*, but failed. The ComP-ComA signaling system is a conserved QS in *Bacillus* genus, and its roles in the development of competence or switch to sporulation are well studied. On the hypothesis that the bacterial pathogenesis against its hosts should be a “social” behavioral mode, we investigated the involvement of ComP-ComA in pathogenesis of *B. nematocida* B16. Our experimental evidences from the assays of Δ*comP* mutant suggested that ComP-ComA participated in the pathogenic processes at least including attractant synthesis and the expressions of virulence proteases. Furthermore, our experimental evidences from transcriptional regulation of virulence proteases confirmed that the transcription factor ComA should directly control the expression of neutral protease Bae16. But it indirectly played the role in the expression of serine protease Bace16 via cis-activating elements DegU, and this data was also validated by the analysis of comparative genomics. Meantime, cis-activating element of the ComA in *B. nematocida B16* was also determined (Figure 5B), similar to the consensus ComA binding sequence in DBTBS TTGCGGNNNNCCGCAA.

Genomic analysis supported the involvement of ComP-ComA signaling system in bacterial pathogenesis in two ways. Firstly, a variety of pathogenic genes were directly regulated by ComA, for example the putative carboxyphosphonoenolpyruvate phosphonomutase (*yqiQ*), NTP pyrophosphohydrolase family protein (*mutT*), NDP-sugar dehydrogenase-like protein (*ywqF*) besides the types of genes listed in our results above. Functionally, most of the target genes have been described to influence infective events such as chemotaxis/mobility, colonizing and defense of pathogens, and destruction of hosts. At the same time, ComA utilized transcriptional network through its impact on the expression of other transcription factors, including degQ-degU system, to participate in infection indirectly. Additionally, although the convergent physiological regulation of similar genes and processes indicated the important and conserved nature of the ComP-ComA signaling system, the distinct difference in regulatory proteins between the saprophytic bacteria *B. subtilis* and the nematotoxic bacteria *B. nematocida* suggest that they are likely associated with pathogenic capability and host environment adaptation.

Conclusively, our current investigation in *B. nematocida* B16 confirmed for the first time the participation and necessity of ComP-ComA signaling system in bacterial pathogenesis.

## Supporting Information

File S1 **It contains: Table S1, Table S2, Table S3, Table S4, and Figures S1-S3.** Figure S1. Analysis to phylogeny (A) and nematocidal activities (B) among B. *Nematocida* and its neighbors in the genus of Bacillus. Our data demonstrated that the model species B. subtilus had little nematocidal activity that was comparable with the negative control of *E. coli*. Figure S2. Homologous modeling predicted high homologies in protein three-dimensional structures of the trans-activating factors CodY (A), AbrB (B), DegU (C), ComA (D) and PurR (E) between B. *Nematocida* and B. subtilus. Green represented the trans-activating factors from B. *Nematocida*; yellow represented the trans-activating factors from B. subtilus.Figure S3. Purification of the heterologously expressed protein ComA of B.nematocida B16. M represented the molecular markers of protein.
(DOC)
