# Supplementary material for: The ComP-ComA Quorum System Is Essential For “Trojan horse” Like Pathogenesis in Bacillus nematocida
Source: PLoS One. 2013 Oct 9;8(10):e76920. doi: 10.1371/journal.pone.0076920 (PMC3793909; doi:10.1371/journal.pone.0076920)
Supplement: File S1 — It contains: Table S1, Table S2, Table S3, Table S4, and Figures S1-S3. Figure S1. Analysis to phylogeny (A) and nematocidal activities (B) among B. Nematocida and its neighbors in the genus of Bacillus. Our data demonstrated that the model species B. subtilus had little nematocidal activity that was comparable with the negative control of E. coli. Figure S2. Homologous modeling predicted high homologies in protein three-dimensional structures of the trans-activating factors CodY (A), AbrB (B), DegU (C), ComA (D) and PurR (E) between B. Nematocida and B. subtilus. Green represented the trans-activating factors from B. Nematocida; yellow represented the trans-activating factors from B. subtilus.Figure S3. Purification of the heterologously expressed protein ComA of B.nematocida B16. M represented the molecular markers of protein. (DOC) [file pone.0076920.s001.doc]

**Figure S1.** Analysis to phylogeny (A) and nematocidal activities (B) among *B. nematocida* and its neighbors in the genus of *Bacillus*. Our data demonstrated that the model species *B. subtilus* had little nematocidal activity that was comparable with the negative control of *E. coli*.

**
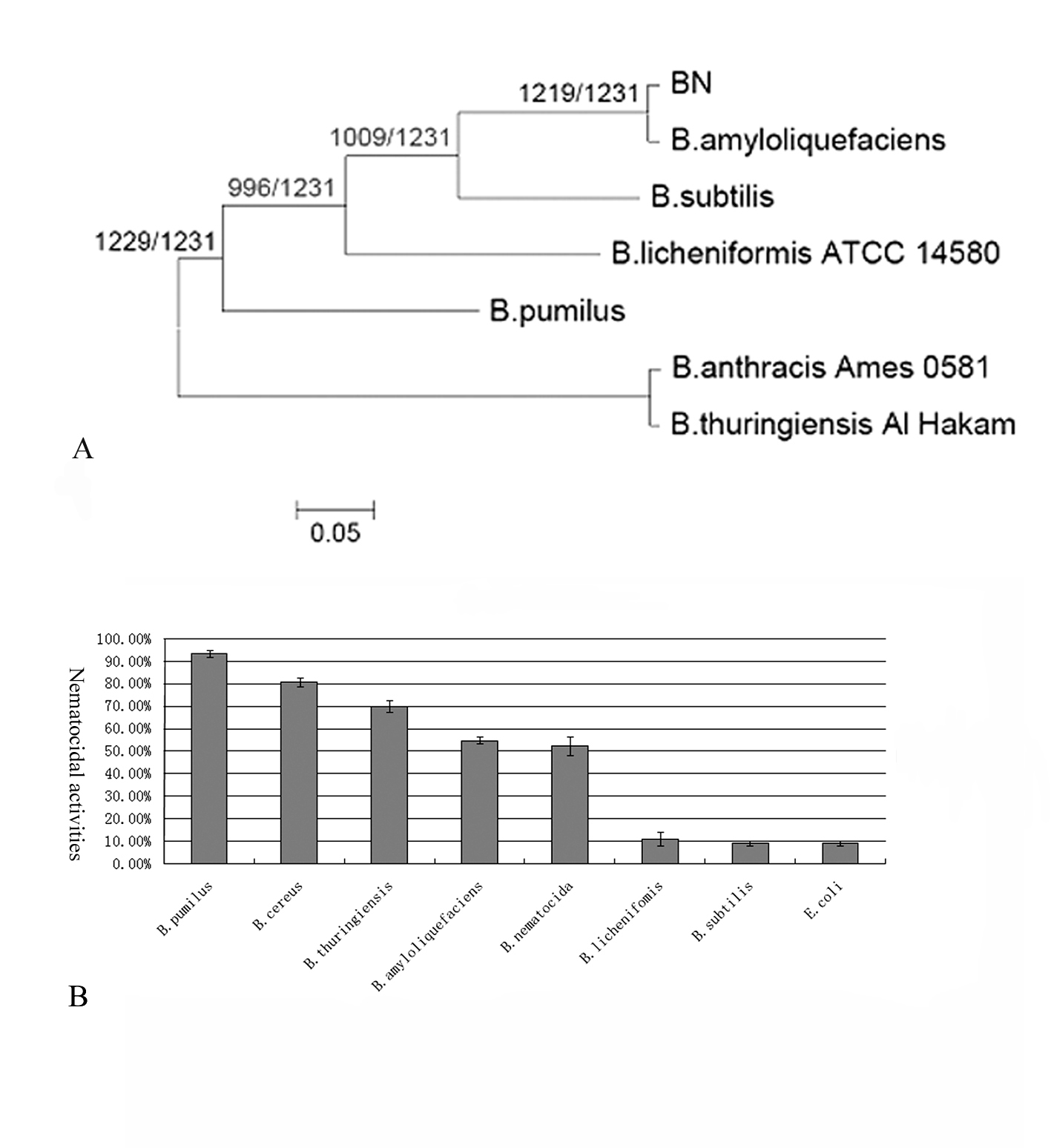
**

**Figure S2.** Homologous modeling predicted high homologies in protein three-dimensional structures of the trans-activating factors CodY (A), AbrB (B), DegU (C), ComA (D) and PurR (E) between *B. nematocida* and *B. subtilus*. Green represented the trans-activating factors from *B. nematocida*; yellow represented the trans-activating factors from *B. subtilus*.

**
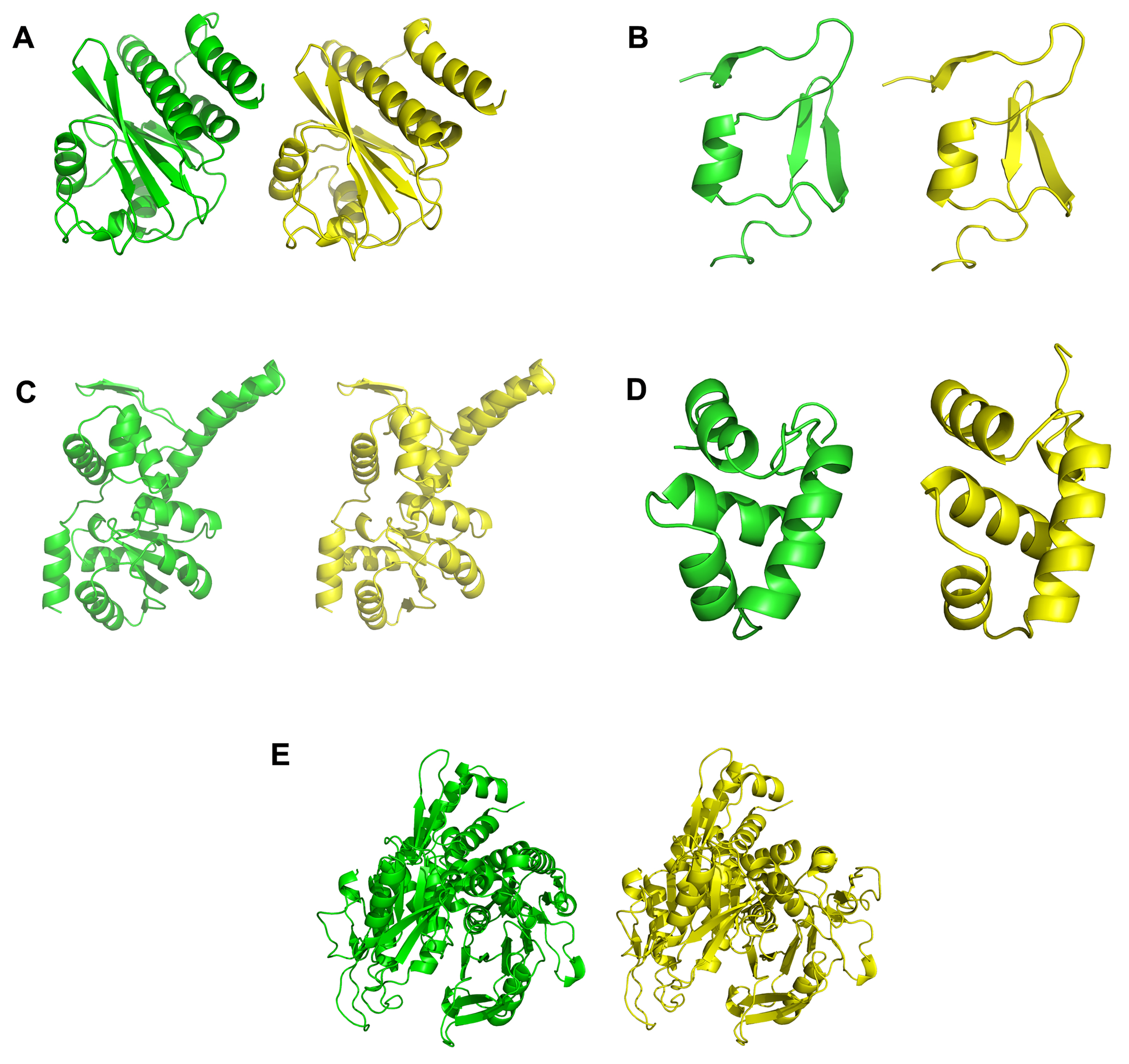
**

**Figure S3.** Purification of the heterologously expressed protein ComA of *B.nematocida* B16. M represented the molecular markers of protein.


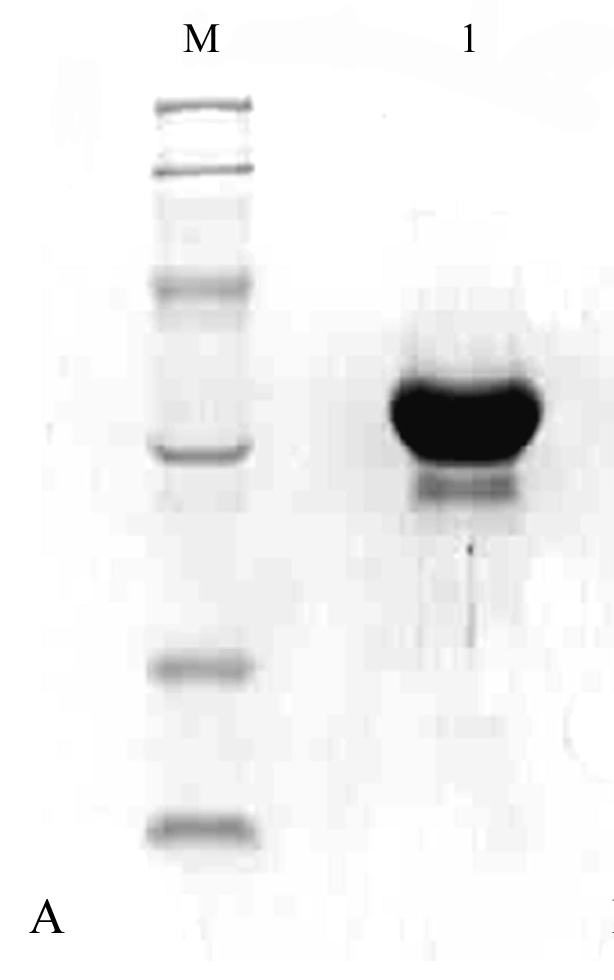


***Table S1 Operons contain upstream ComA binding sites in*** B. nematocida

| **Operon** | **Binding site** |
| --- | --- |
| csfB* | TGCGGGCCCCCGTCA |
|  | TGCGTGAAGCTGGAA |
| ybaN | TGCGGGCCCCCGTCA |
|  | TGCGTGAAGCTGGAA |
| srfAA* | TGCGGCACACCGAAA |
|  | TGCGGGATGCCGCAA |
|  | TGCGGCATCCCGCAA |
| BN_2861 | TGCGTGAAGCTGGAA |
|  | TGCGGGCCCCCGTCA |
| gcp* ydiD* ydiC* ydiB* thiL* | TGCGTGAAGCTGGAA |
|  | TGCGGGCCCCCGTCA |
| yrzI | TGCGGGGAGCTGCGA |
|  | TGCGGGCAGACTAAA |
| BN_1032 BN_1033 | TTCGACATGCCGCAA |
|  | TGCGGCATGTCGAAA |
| YwqJA | TTCGACATGCCGCAA |
|  | TGCGGCATGTCGAAA |
| ykuK | TCCGGTCTTCCGCAA |
|  | TGCGGAAGACCGGAA |
| BN_0730 | TGCGCAAAGCCGGAA |
|  | GGCGGGTTGCCGATT |
| rtp | TTCGGGTTGCGGAAA |
| yfkD yfkE | TTCGGGCTGCCGCGG |
| yfkF | TTCGGGCTGCCGCGG |
| yosR | TTCGGGAAGCCAAAA |
| ctaG ctaF ctaE ctaD ctaC | TGCTTCCAGCCGAAA |
| bae16 | TGCTGGCCGCCGGAA |
| ylaD | TGCTGGCCGCCGGAA |
| BN_3613 | TGCGTGTTGCGGAAT |
| ywhA | TGCGTGTTGCGGAAT |
| BN_0596 | TGCGTGTTCCTGCCA |
| BN_0746 *BN_0747* | TGCGTGTTCCTGCCA |
| BN_2832 | TGCGTGTTCCTGCCA |
| yndH yndJ | TGCGTGATCCGTCAA |
| degQ* ComQ *BN_0650* comP comA yuxO | TGCGTGACACCGCAA |
| yuzC* | TGCGTGACACCGCAA |
| sdpC | TGCGTCCGGCCGTAA |
| ybgB | TGCGTCCGGCCGTAA |
| zwf | TGCGGTTTACGGAAA |
| rnz *rpmG* | TGCGGTTTACGGAAA |
| BN_2649 BN_2650 | TGCGGTCTGCCTGAA |
| yrvM* | TGCGGGCTCCCAAAT |
| BN_2458 | TGCGGGCGTCCGCCA |
| BN_1036 | TGCGGGCAGCCTACA |
| yneN | TGCGGGAGGCCTTAA |
| BN_2517 | TGCGGGAACGCTCAA |
| BN_3602 BN_3603 BN_3604 | TGCGGATTTCCGAAA |
| phrA* rapA* | TGCGGATATCCGAAA |
| yjoA | TGCGGATATCCGAAA |
| yosT | TGCGGAATTCCTAAA |
| phrC* rapC* | TGCGCTCTGCCGAAA |
| yqiH yqiI yqiK mmgA mmgB mmgC mmgD prpD yqiQ | TGCGCTCTCCCGAAT |
| BN_0884 | TGCGCGATGTCGAAA |
| BN_0727 *BN_0728* | TGCGATAATCCGAAA |
| BN_2848 | TGCGATAATCCGAAA |
| ycsA | TGCCGTAAACCGAAA |
| ydhC | TGCCGTAAACCGAAA |
| parE parC | TGCCGGCCATCGCAA |
| xkdR | TGCAGTATACCGAAA |
| BN_2857 BN_2858 | TGCAGGTAGCCGATA |
| BN_0591 *BN_0592* | TGCAGGCGGCCGCAC |
| YybE | TGAGGGCTTCCGGAA |
| BN_0882 | TGAGGGCTGCCGCTG |
| BN_0729 | TACGTGATCCCGGAA |
| yrhH | TACGGGTTGCCGTAG |
| yosV *BN_1900* | TACGGGATACAGAAA |
| ywdI | GGCGGTATGCCGCCA |
| ywqG*ywqF | GGCGGAATGCCGGAA |
| BN_1024 | GGCAGGACTCCGCAA |
| ydaP mutT ydaO | CGCGTGATGCCGCTT |
| dbpA BN_3768 *BN_3769* *BN_3770* yxiI | CGCGGGAGGCAGAAA |
| cotF | CGCGGACAGCCGCAA |
| rplI yybT yybS | CGCGGACAGCCGCAA |
| yojH yojI | CGCGCGATGCAGAAA |
| yojJ | CGCGCGATGCAGAAA |
| hemD | AGCGTGTAGTCGCAA |
| yflP citT citS *BN_2967 YwpE* | AGCGGGTTACGGAAA |
| BN_0233 | AGCGGGGCGCTGAAA |
| BN_1729 | AGCGGGAAACGGAAA |
| ynaE | AGCGGGAAACGGAAA |
| motA motB | AGCGGCTCCCCGAAA |
| ywrK | AGAGGGCCGCCGAAA |
| yqaH *BN_1005* yqaI *BN_1007* | AACGGGACGCCGAAA |

***Table S2 The candidate target genes containing upstream ComA binding sites in*** B. nematocida

| **Gene name** | **Locus** | **Prediction** | **COG category** | **Pathway** |
| --- | --- | --- | --- | --- |
| zwf | BN_1380 | Glucose-6-phosphate 1-dehydrogenase | COG0364G | ko00030 ko00480 |
| yybT | BN_3889 | conserved membrane protein | COG3887T |  |
| yybS | BN_3890 | Predicted membrane protein | COG4241S |  |
| YybE | BN_3901 | positive Regulator of yybF (LysR family) |  |  |
| yxiI | BN_3771 | function unknown and unique |  |  |
| ywrK | BN_3471 | Na+/H+ antiporter NhaD and related arsenite permeases | COG1055P |  |
| YwqJA | BN_1031 |  |  |  |
| ywqG* | BN_3490 | conserved protein | COG3878S |  |
| ywqF | BN_3491 | Predicted UDP-glucose 6-dehydrogenase | COG1004M | ko00040 ko00053 ko00500 ko00520 |
| YwpE | BN_2968 | Sortase (surface protein transpeptidase) | COG3764M |  |
| ywhA | BN_3612 | Transcriptional regulators | COG1846K |  |
| ywdI | BN_3664 | function unknown and unique |  |  |
| yuzC* | BN_0647 | conserved protein |  |  |
| yuxO | BN_0653 | Possibly involved in aromatic compounds catabolism | COG2050Q |  |
| yrzI | BN_1153 | function unknown and unique |  |  |
| yrvM* | BN_1117 | Dinucleotide-utilizing enzymes involved in molybdopterin and thiamine biosynthesis | COG1179H |  |
| yrhH | BN_1154 | SAM-dependent methyltransferases | COG0500QR |  |
| yqiQ | BN_1356 | PEP phosphonomutase and related enzymes | COG2513G |  |
| yqiK | BN_1350 | Glycerophosphoryl diester phosphodiesterase | COG0584C | ko00564 |
| yqiI | BN_1349 | N-acetylmuramoyl-L-alanine amidase | COG0860M |  |
| yqiH | BN_1348 | similar to B. subtilis LytA |  |  |
| YqbO | BN_0747 | similar to phage-related protein, SKIN protein |  |  |
| yqaI | BN_1006 | SKIN protein |  |  |
| yqaH | BN_1004 | SKIN protein |  |  |
| yosV | BN_1899 | SP-beta protein |  |  |
| yosT | BN_1648 | DNA gyrase inhibitor | COG3449L |  |
| yosR | BN_1901 | Thiol-disulfide isomerase and thioredoxins | COG0526OC |  |
| yojJ | BN_1687 | Uncharacterized conserved protein | COG1624S |  |
| yojI | BN_1686 | Na+-driven multidrug efflux pump | COG0534V |  |
| yojH | BN_1685 | regulator of sigma-B | COG1366T |  |
| yneN | BN_1760 | Thiol-disulfide isomerase and thioredoxins | COG0526OC |  |
| yndJ | BN_1783 | function unknown and unique |  |  |
| yndH | BN_1782 | function unknown and unique |  |  |
| ynaE | BN_1730 | conserved protein |  |  |
| ylaD | BN_2258 | Predicted integral membrane protein | COG5660S |  |
| ykuK | BN_2326 | Uncharacterized protein conserved in bacteria | COG1978S |  |
| yjoA | BN_2475 | Uncharacterized protein conserved in bacteria | COG2318S |  |
| yflP | BN_2964 | Uncharacterized protein conserved in bacteria | COG3181S |  |
| yfkF | BN_2931 | Multidrug-efflux transporter | COG0477GEPR |  |
| yfkE | BN_2930 | Ca2+/H+ antiporter | COG0387P |  |
| yfkD | BN_2929 | conserved protein |  |  |
| ydiD* | BN_3108 | Acetyltransferases | COG0456R |  |
| ydiC* | BN_3109 | Similar to glycoprotein endopeptidase | COG1214O |  |
| ydiB* | BN_3110 | Predicted ATPase or kinase | COG0802R |  |
| ydhC | BN_3129 | Transcriptional regulators | COG1802K |  |
| ydaP | BN_3268 | Thiamine pyrophosphate-requiring enzymes | COG0028EH | ko00620 |
| ydaO | BN_3270 | Amino acid transporters | COG0531E |  |
| ycsA | BN_3130 | Isocitrate/isopropylmalate dehydrogenase | COG0473CE |  |
| ybgB | BN_0542 | Predicted integral membrane protein | COG5658S |  |
| ybaN | BN_0158 | Predicted xylanase/chitin deacetylase | COG0726G |  |
| xkdR | BN_2455 | PBSX prophage protein |  |  |
| thiL* | BN_3111 | Thiamine monophosphate kinase | COG0611H | ko00730 |
| srfAA | BN_3357 | Surfactin synthetase subunit 1 | COG1020,COG1020,COG1020Q |  |
| sdpC | BN_0543 |  |  |  |
| rtp | BN_1842 | replication termination protein |  |  |
| rpmG | BN_1382 | Ribosomal protein L33 | COG0267J | ko03010 |
| rplI | BN_3888 | Ribosomal protein L9 | COG0359J | ko03010 |
| rnz | BN_1381 | Metal-dependent hydrolases of the beta-lactamase superfamily III | COG1234R | ko03013 |
| rapC* | BN_3323 | Response regulator aspartate phosphatase | COG0457R |  |
| rapA* | BN_2474 | response regulator aspartate phosphatase. prevents sporulation by dephosphorylating Spo0F-P (and thus the phosphorelay) | COG0457R |  |
| prpD | BN_1355 | Uncharacterized protein involved in propionate catabolism | COG2079R | ko00640 |
| phrC | BN_3322 | regulator of the activity of phosphatase RapC and competence and sporulation stimulating factor (CSF) |  |  |
| phrA* | BN_2473 | phosphatase rapA inhibitor |  |  |
| parE | BN_1769 | Topoisomerase IV subunit B | COG0187L |  |
| parC | BN_1770 | Topoisomerase IV subunit A | COG0188L |  |
| bae16 | BN_2259 | Zinc metalloprotease (elastase) | COG3227E |  |
| mutT | BN_3269 | NTP pyrophosphohydrolases including oxidative damage repair enzymes | COG0494LR |  |
| motB | BN_2368 | Chemotaxis motB protein (motility protein B) | COG1360N | ko02030 ko02040 |
| motA | BN_2367 | Chemotaxis motA protein (motility protein A) | COG1291N | ko02020 ko02030 ko02040 |
| mmgD | BN_1354 | Citrate synthase | COG0372C | ko00020 ko00630 |
| mmgC | BN_1353 | Acyl-CoA dehydrogenases | COG1960I |  |
| mmgB | BN_1352 | 3-hydroxyacyl-CoA dehydrogenase | COG1250I | ko00650 ko00360 ko00362 |
| mmgA | BN_1351 | Acetyl-CoA acetyltransferase | COG0183I | ko00620 ko00630 ko00640 ko00650 ko00720 ko00071 ko00072 ko00280 ko00310 ko00380 ko00900 ko00362 ko02020 |
| hemD | BN_2489 | Uroporphyrinogen-III synthase | COG1587H | ko00860 |
| gcp* | BN_3107 | O-sialoglycoprotein endopeptidase, eesential protein | COG0533O |  |
| degQ* | BN_0648 | degradation enzyme regulation protein |  |  |
| dbpA | BN_3767 | Superfamily II DNA and RNA helicases | COG0513LKJ |  |
| ctaG | BN_2238 | Predicted membrane protein | COG3336S |  |
| ctaF | BN_2239 | cytochrome caa3 oxidase subunit IV | COG3125C | ko00190 |
| ctaE | BN_2240 | cytochrome caa3 oxidase subunit III | COG1845C | ko00190 ko00910 |
| ctaD | BN_2241 | cytochrome caa3 oxidase subunit I | COG0843C | ko00190 ko00910 |
| ctaC | BN_2242 | cytochrome caa3 oxidase subunit II | COG1622,COG2857 | ko00190 ko00910 |
| csfB* | BN_0025 | sigma-F transcribed gene |  |  |
| cotF | BN_3891 | Spore coat protein | COG5577M |  |
| ComQ | BN_0649 | Transcriptional regulator of comG and srfA | COG0142H |  |
| comP | BN_0651 | two-component sensor histidine kinase. cognate response regulator is comA | COG4585T | ko02020 |
| comA | BN_0652 | two-component response regulator. cognate sensor kinase is comP | COG2197TK | ko02020 |
| citT | BN_2965 | Response regulator of citrate/malate metabolism | COG4565KT | ko02020 |
| citS | BN_2966 | two-component sensor histidine kinase involved in the response to the environmental Mg-citrate complex. cognate response regulator is CitT | COG3290T | ko02020 |
| blt | BN_2650 | Multidrug-efflux transporter | COG0477GEPR |  |
| - | BN_0884 |  |  |  |
| - | BN_2858 |  |  |  |
| - | BN_1729 |  |  |  |
| - | BN_1033 |  |  |  |
| - | BN_2458 |  |  |  |
| - | BN_3768 |  |  |  |
| - | BN_3602 |  |  |  |
| - | BN_3603 |  |  |  |
| - | BN_3769 |  |  |  |
| - | BN_3770 |  |  |  |
| - | BN_3604 |  |  |  |
| - | BN_2857 |  |  |  |
| - | BN_2832 |  |  |  |
| - | BN_2517 |  |  |  |
| - | BN_1005 |  |  |  |
| - | BN_1007 |  |  |  |
| - | BN_0746 |  |  |  |
| - | BN_0730 |  |  |  |
| - | BN_0650 |  |  |  |
| - | BN_0596 |  |  |  |
| - | BN_2967 |  |  |  |
| - | BN_1900 |  |  |  |
| - | BN_1024 |  |  |  |
| - | BN_0882 |  |  |  |
| - | BN_0727 |  |  |  |
| - | BN_0591 |  |  |  |
| - | BN_0592 |  |  |  |
| - | BN_1032 | FOG: TPR repeat | COG0457R |  |
| - | BN_3613 | Amino acid transporters | COG0531E |  |
| - | BN_2861 | Integrase | COG0582L |  |
| - | BN_2848 | dUTPase | COG0756F | ko00240 |
| - | BN_0728 | dUTPase | COG0756F | ko00240 |
| - | BN_0233 | ABC-type polysaccharide/polyol phosphate transport system, ATPase component | COG1134GM | ko02010 |
| - | BN_2649 | Transcriptional regulator | COG1309K |  |
| - | BN_1036 | Site-specific recombinases, DNA invertase Pin homologs | COG1961L |  |
| - | BN_0729 | Uncharacterized protein conserved in bacteria | COG3584S |  |

*represents the genes that were predicted the target genes in both *B. nematocida* and *B. subtilus.*

The genes underlined represented that there were not the othorlogs identified in *B. subtilus* or little similarities had been found between the genes of the two specieses.

**Table S3** Strains and plasmids used in this study

| Strain or plasmid | Description | Reference of source |
| --- | --- | --- |
| Strains |  |  |
| ***B. nematocida* B16** | Wild type | laboratory stock |
| BC | Δ*comP* mutant in *B. nematocida* B16 | this work |
| BA1 | *B.nematocida* B16 amyE::P_bace16-499bp_-lacZ(Cm^r^) | this work |
| BA2 | *B.nematocida* B16 amyE::P_bace16-422bp_-lacZ(Cm^r^) | this work |
| BA3 | *B.nematocida* B16 amyE::P_bace16-280bp_-lacZ(Cm^r^) | this work |
| BA4 | *B.nematocida* B16 amyE::P_bace16-93bp_-lacZ(Cm^r^) | this work |
| BA5 | *B.nematocida* B16 amyE::P_bace16+65bp_-lacZ(Cm^r^) | this work |
| BN1 | *B.nematocida* B16 amyE::P_bae16-509bp_-lacZ(Cm^r^) | this work |
| BN2 | *B.nematocida* B16 amyE::P_bae16-218bp_-lacZ(Cm^r^) | this work |
| BN3 | *B.nematocida* B16 amyE::P_bae16-148bp_-lacZ(Cm^r^) | this work |
| ***E.coli*** |  |  |
| BL21 | *E. coli* strain for heterologously expressing protein | laboratory stock |
| BL-comA | BL21 heterologously expressing ComA of B16 | this work |
| DH5α | Cloning strain | laboratory stock |
|  |  |  |
| **Plasmids** |  |  |
| Pcp115 | integration vector for Gram-positive bacterium | obtained from BGSC |
| *P*cp115Δ*comP* | integration vector for constructing Δ*comP* mutant in B16 | this work |
| pDG148 | expressional plasmid in *B. subtilis* | obtained from BGSC |
| pDG*comP* | complemente *comP* in Δ*comP* mutant | this work |
| Pis284 | insertion vector to amyE, chloramphenicol resistance, lacZ | present from **Mitsuo Ogura** |
| pA1 | Pis284 carrying positions -499 to +170 of the bace16 promoter | this work |
| pA2 | Pis284 carrying positions -422 to +170 of the bace16 promoter | this work |
| pA3 | Pis284 carrying positions -280 to +170 of the bace16 promoter | this work |
| pA4 | Pis284 carrying positions -93 to +170 of the bace16 promoter | this work |
| pA5 | Pis284 carrying positions +65 to +170 of the bace16 promoter | this work |
| pN1 | Pis284 carrying positions -509 to +156 of the bae16 promoter | this work |
| pN2 | Pis284 carrying positions -218 to +156 of the bae16 promoter | this work |
| pN3 | Pis284 carrying positions -148 to +156 of the bae16 promoter | this work |
| pET30a | expressional plasmid in *E. coli* | laboratory stock |
| pET30-comA | recombinant expressional plasmid for expressing ComA of B16 | this work |

**Table S4 The** **primers used in this study**

| Primer | Nucleotide sequence (5′→3′) |
| --- | --- |
| comP1 (for) | GAATTCAGCCATACCGGGTTTGTCTC |
| comP2 (rev) | CTGCAGCGCAATGAAAGTAGGAGAACG |
| comP3 (for) |  |
| comP4 (rev) | AGA AGACAGTCATAAGTGCGG |
| O1 (for) | AGCAGTAGGGAATCTTCCGCAATG |
| O2 (rev) | ACGACACGAGCTGACGACAACCA |
| E1 (for) | AAACAGCCAACTTGAACTATGA |
| E2 (rev) | GCTTGATGACGGAGTGAGAT |
| J1 (for) | GTCCGTGTATTAGGCGGTGCG |
| J2 (rev) | GTGTTTGTCCAGTTCGGGTGC |
| A1(for) | GAATTCtcctctttcatttttccgc |
| A2 (for) | GAATTCtggtttcacagcttttctccg |
| A3 (for) | GAATTCtggaatcaaaccgttcgac |
| A4 (for) | GAATTCccatgctatacaattaatcc |
| A5 (for) | GAATTCGATCAGTTTGCTGTTTGCTT |
| ARV(rev) | AAGCTT CATCGTGCTCATTGTCTG |
| N1 (for) | GAATTCtgtttcagctgcattctcg |
| N2 (for) | GAATTCgtctgattcaacacgtgcctc |
| N3 (for) | GAATTCtatcaattcatcagcggag |
| NRV (rev) | AAGCTTGCTTGATTGCTTTGTCACT |
| Q1^a^ (for) | ccgatgctggccgccggaagccttt |
| RQ1^a^ (rev) | AAAGGCTTCCGGCGGCCAGCATCGG |
| LQ1 (for) | ccgatgctggccgccggaagccttt |
| LQ2 (rev) | AAAGGCTTCCGGCGGCCAGCATCGG |
| ComA (for) | GGATCC atgaaaaagatactagtga |
| ComA (rev) | AAGCTT TTATAGTACGTTATCTGACT |

^a^X: biotin attached to the nucleotide at the 5′end.
